# Cross-reactivity and sequence similarity between microbial transglutaminase and human tissue antigens

**DOI:** 10.1038/s41598-023-44452-5

**Published:** 2023-10-16

**Authors:** Aaron Lerner, Carina Benzvi, Aristo Vojdani

**Affiliations:** 1 Chaim Sheba Medical Center, The Zabludowicz Research Center for Autoimmune Diseases, Tel Hashomer, Israel; 2 Ariel University, Ariel, Israel; 3 Immunosciences Lab., Inc., Los Angeles, CA 90035 USA

**Keywords:** Immunology, Diseases, Gastroenterology, Pathogenesis

## Abstract

Microbial transglutaminase (mTG) is a bacterial survival factor, frequently used as a food additive to glue processed nutrients. As a result, new immunogenic epitopes are generated that might drive autoimmunity. Presently, its contribution to autoimmunity through epitope similarity and cross-reactivity was investigated. Emboss Matcher was used to perform sequence alignment between mTG and various antigens implicated in many autoimmune diseases. Monoclonal and polyclonal antibodies made specifically against mTG were applied to 77 different human tissue antigens using ELISA. Six antigens were detected to share significant homology with mTG immunogenic sequences, representing major targets of common autoimmune conditions. Polyclonal antibody to mTG reacted significantly with 17 out of 77 tissue antigens. This reaction was most pronounced with mitochondrial M2, ANA, and extractable nuclear antigens. The results indicate that sequence similarity and cross-reactivity between mTG and various tissue antigens are possible, supporting the relationship between mTG and the development of autoimmune disorders 150W.

## Introduction

Genetic predisposition is pivotal for autoimmune diseases (ADs) development, but environmental factors are necessary for their clinical evolvement^1–4^. Pending on their association with various ADs, they include: hygiene and diet^5^, food processed additives^6–9^, trace elements^10^, enteric microbial peptides^11^, multiple infectious agents^3,12–15^, various vaccines^16,17^, toxic agents or food products^4^ and recently the checkpoint inhibitors^18,19^. In fact, many of those environmental factors were cited as part of the autoimmune/inflammatory syndrome induced by adjuvants (ASIA)^1,20,21^. Zooming into the gastrointestinal tract (GIT), many of the above-mentioned environmental factors inhabit the enteric lumen and are associated with local or peripheral ADs^3–13,22^.

Many processes were described to operate in the human GIT driving gut-originated autoimmunity. The most reported one is increased gut permeability resulting in leaky gut syndrome^6,23–25^. Among others are posttranslational modification of naïve proteins^26^, dysbiosis and its harmful mobilome^23,27^, horizontal gene transfer^28^, or many immunogenic nutritional compounds, such as gluten^4,6–9,22,27^. All those enteric events irradiate peripherally and might induce systemic autoimmunity^29^.

Indeed, some of those luminal events are blamed to increase the worldwide incidence of ADs^23,30^. Among the various mechanisms that drive autoimmunity, molecular mimicry is the most reported^11,16,31,32^. Actually, SARS-CoV-2-associated autoimmunity is suggested to operate through molecular mimicry with self-epitopes^33,34^.

Transglutaminases are an extensive natural enzymatic family that catalyze the formation of isopeptide bonds by post-translational modification of proteins. In fact, they are considered nature's biological glues^35^. In the presence of an acyl donor and an acyl acceptor they cross-link the corresponding protein to form a protease-indigestible high molecular mass protein^36,37^. The accumulated linked complexes can be deposited in various tissues and organs and are involved in multiple human chronic diseases. Inflammatory, cancerous, metabolic, neurodegenerative and ADs^7,22,27,38,39^ are some examples.

The present study is focused on the frequently consumed processed food additive, namely, microbial transglutaminase (mTG). It is considered a natural family member of the transaminases. Despite having a very low sequence similarity and having a much lower molecular weight, the sequence similarity is much higher on their active site^8^. Functionally, it imitates the transglutaminases’ cross-linking activity; all of them can deamidate or transamidate their substrates^6–9,22,27,29^. In recent years, the mTG enzyme has been reported to functionally join tissue transglutaminase (tTG), which is the autoantigen of celiac disease (CD)^6,8,36,37^. In fact, mTG has been suggested as a new environmental factor in CD^8,9,40–45^, other ADs^22,27,45^ and as even being involved in the induction of neurodegenerative diseases^7,22,39^. Presently, a new aspect of the potential harmful effects of the mTG enzyme in the induction of ADs are being described. The transcytosis of the mTG to the sub-epithelial compartment^46^, the immune reactions against the mTG-gliadin complexes in CD patients^8,9,22,27,39–50^ and the most recently described resistance of the mTG to oxidative stress in CD^51^ prompted us to conduct the present study. The cross-reactivity and sequence similarity between mTG and human epitopes have never been explored. The present hypothesis is that cross-reactive antibodies and sequence similarity between the mTG and human self-epitopes further reinforce the relationship of molecular mimicry between the mTG enzyme and the induction of chronic inflammatory, autoimmune and neurodegenerative diseases. Moreover, epitope sharing between these self-proteins and gluten might suggest the involvement of mTG cross-linked gluten complexes in non-celiac chronic conditions.

## Results

### Cross-reactive polyclonal antibodies to human proteins

The application of affinity-purified polyclonal antibody against mTG to many human tissue antigens resulted in different degrees of reactivity. 57 antigens resulted in ODs of around 0.16 + 0.12, which was very similar to the ELISA background or negative controls.

The cutoff was established based on the optical densities of these 57 antigens, 0.16 + 0.12 = 0.28. Two antigens, cardiolipin and TG6, showed ODs very close to the cutoff point of 0.28. Cardiolipin had an OD of 0.26 and a *p* value of 0.1, which was not significant. TG6 had an OD of 0.29 and a *p* value of 0.0004, which was very significant. The higher the OD is above the cutoff point, the more statistically significant the value was. For instance, the next antigen over the cutoff point, somatotropin, had an OD of 0.45 and a *p* value of 0.0001.

**Figure 1:**
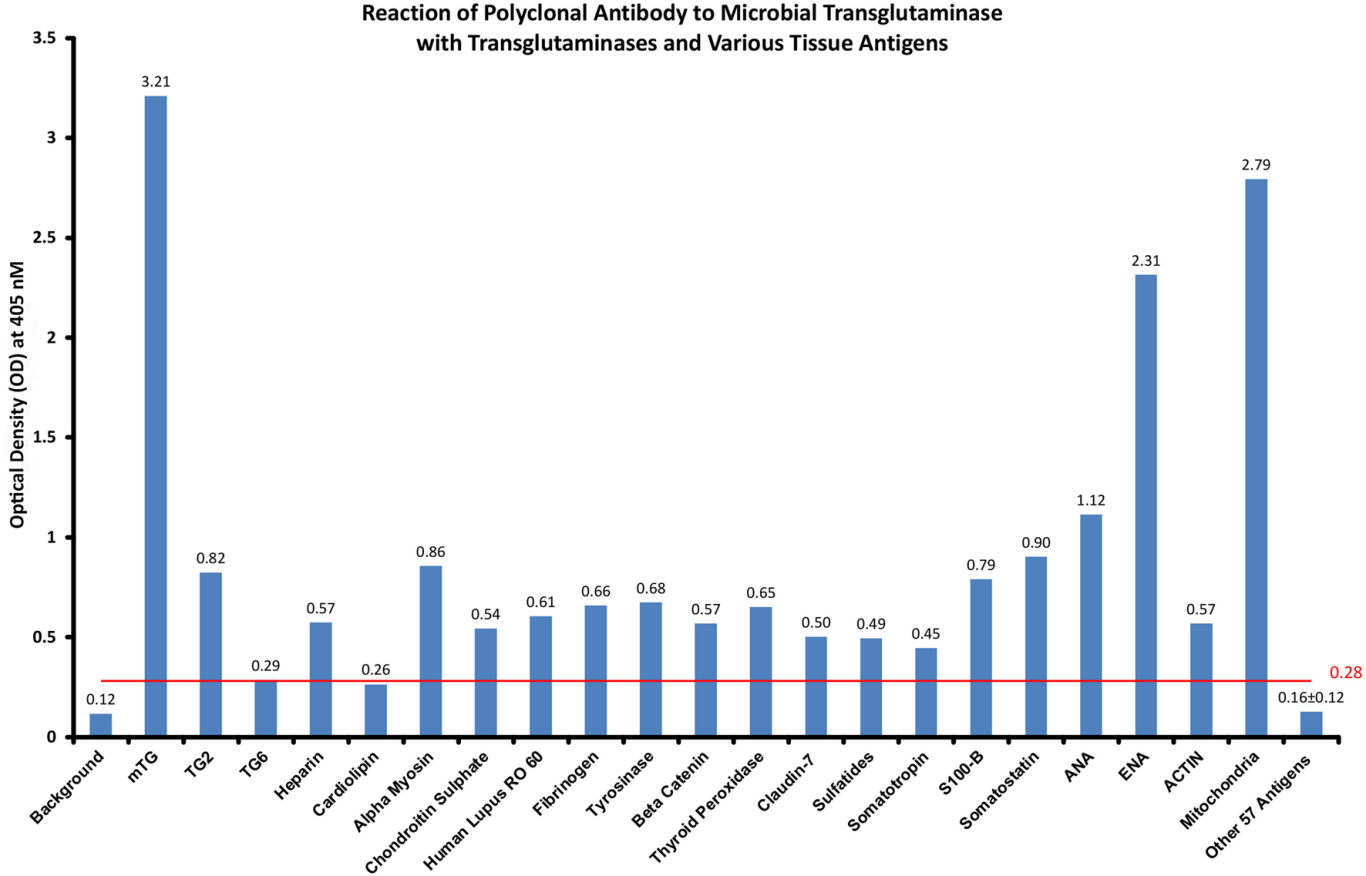
Rabbit polyclonal antibody to mTG and its reaction to human tissue components expressed as ELISA ODs. This reaction was performed in duplicate, and variation in the ODs between the duplicate wells was less than 7%. As shown, the reaction of this antibody with mitochondrial M2 antigen and ENA resulted in the highest ODs.

Mitochondria (M2) and ENA had the highest ODs. Although lesser ODs were detected for ANA, TG2, TG6, heparin, α-myosin, chondroitin sulfate, Lupus RO-60, fibrinogen, tyrosinase, β catenin, thyroid peroxidase, claudin 7, sulfatides, somatotropin, S100B, somatostatin and actin, their *p* values were still very, very significant (Fig. 1).

### Cross reactive mouse monoclonal antibodies to human proteins

The application of affinity-purified mouse monoclonal antibody against mTG to many human tissue antigens resulted in different degrees of reactivity. 67 antigens resulted in ODs of around 0.15 + 0.09, which was very similar to the ELISA background or negative controls. The cutoff was established based on the optical densities of these 67 antigens, 0.15 + 0.09 = 0.24. TG6 had an OD below the cutoff point of 0.24, with an OD of 0.16 and a *p* value of 0.05, which was not significant. Mitochondria had an OD of 0.33 and a *p* value of 0.00007, which was very significant. The higher the OD is above the cutoff point, the more statistically significant the value was. For instance, the next antigen over the cutoff point, TG3, had an OD of 0.38 and a *p* value of 0.0000003.

**Figure 2:**
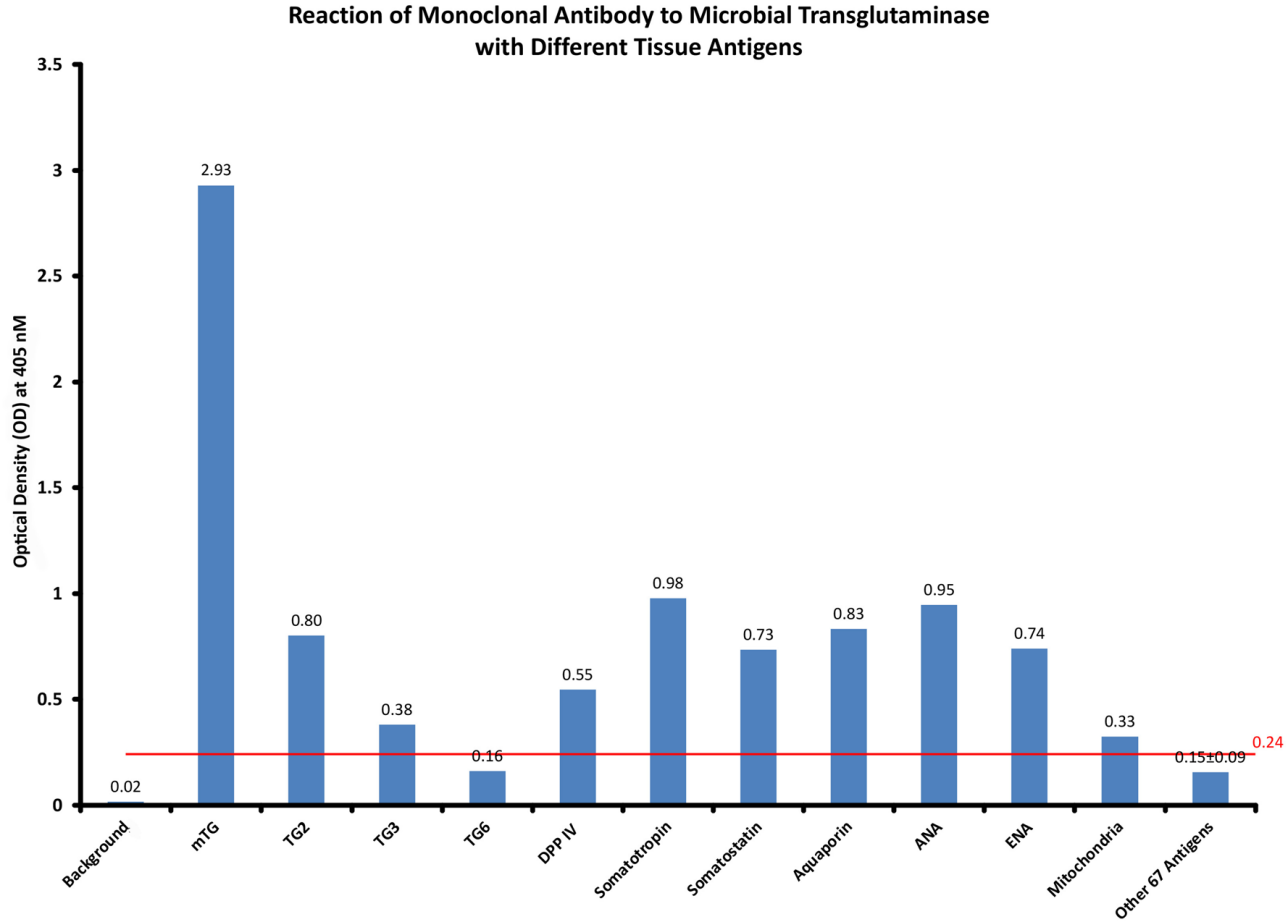
Mouse monoclonal antibody against mTG and its reaction to human tissue components expressed as ELISA ODs. This reaction was performed in duplicate, and variation in the ODs between the duplicate wells was less than 7%. *DPP IV* Dipeptidyl peptidase IV.

Somatotropin and ANA had the highest ODs. Although lesser ODs were detected for TG2, DPP IV, somatotropin, somatostatin, aquaporin, ANA, and ENA, their *p* values were still very, very significant (Fig. 2).

### Molecular similarity between mTG and human immune epitopes

Out of 67,000 epitopes of human tissue antigens, 60 were detected to share significant homology with sub-sections of mTG protein. Out of those, six pairs of similar sequences were detected between human epitopes derived from cross-reactive antigens and mTG sequences that were considered as immunogenic with a strong binding affinity to at least one of the HLA-I and HLA-II alleles. The human epitopes were derived from tissue antigens that are implicated in 10 ADs: rheumatoid arthritis (RA), ankylosing spondylitis (AS), autoimmune atherosclerosis (AIAS), psoriatic arthritis (PA), autoimmune thyroiditis (AIT), Sjogren's syndrome (SS), primary biliary cholangitis (PBC), type 1 diabetes mellitus (T1DM), multiple sclerosis (MS), and autoimmune uveitis (AU).

Table 1 presents sequence similarities driven by six antigens. Four antigens relate to RA, three to T1DM, two to SS, and one relates to AIAS, PBC, MS, PA, AU, AIT, and AS. The similarity of paired sequences is displayed in red, (human on top of mTG), and the isolated amino acid (AA) mismatches are marked in black. The alignment cut-off was kept at a minimum of seven identical AAs, and peptide length > 12 AAs. The resulting human sequences are presented in Table 2 highlighting the antigens’ functionality and their implications in various ADs.

**Table 1:** Sequence similarity between mTG protein and self-antigens related to ADs.

Human protein (autoimmune-related Disease) ^1^	Human	Ln	Identity	Similarity
	mTG			
Fibrinogen alpha chain (AIAS, AS, RA, PA)	PSRGKSSSYSKQF	13	8/13	9/13
	PSRMKAVIYSKHF			
Histone H1.2 (AIT, SS)	SETAPAAPAAAP	12	7/12	10/12
	NESAPAASSAGP			
"Dihydrolipoyllysine-residue acetyltransferase component of pyruvate dehydrogenase complex, mitochondrial (DLST)” (PBC, SS, T1DM)	AATASPPTPSAQAP	14	7/14	8/14
	APAASSAGPSFRAP			
"Creatine kinase S-type, mitochondrial (mtCK)” (RA, T1DM)	REVENVAITALE	12	7/12	8/12
	REVASVMNRALE			
Dimethyladenosine transferase 2, mitochondrial (TRMT10C) (MS, RA)	LKVVGMFPSRGEKRA	15	7/15	7/15
	LCTAGFMPSAGEAAA			
Cytochrome c1, heme protein, mitochondrial (AU, RA, T1DM)	AANNGALPPDLSY	13	7/13	8/13
	AADNGAGEETKSY			

^1^Autoimmune diseases: rheumatoid arthritis (RA), ankylosing spondylitis (AS), autoimmune atherosclerosis (AIAS), psoriatic arthritis (PA), autoimmune thyroiditis (AIT), Sjogren's syndrome (SS), primary biliary cholangitis (PBC), type 1 diabetes mellitus (T1DM), multiple sclerosis (MS), and autoimmune uveitis (AU), Length (Ln).

**Table 2:** The functionality of identified antigens and their corresponding ADs.

Antigen potential function/pathogenesis	ADs^1^	References
Fibrinogen alpha chain (UniProt:P02671)		
Plays a role in blood coagulation. It is synthesized in the liver and is released into the bloodstream where it circulates in an inactive form. When blood vessels are damaged and start to bleed, fibrinogen is converted into fibrin, a key component in the formation of a blood clot. Fibrin forms a network of fibers that trap red blood cells and platelets at the site of injury, forming a temporary barrier that stops the bleeding	AIAS AS PA RA SLE	^52–57^
Histone H1.2 (UniProt:P16403)		
Component of chromatin that plays a role in regulating gene expression and cellular processes Antibodies against histones have been associated with various ADs	AIT SS SLE PBC	^58,59^
Dihydrolipoyllysine-residue acetyltransferase component of pyruvate dehydrogenase complex, mitochondrial (DLST, UniProt:P10515)		
An enzyme that is involved in the regulation of energy metabolism in cells Autoantibodies against DLST have been found in some individuals with ADs PBC patients have been characterized to have autoreactive T-cell and B-cell responses directed at self-PDC-E2. The diagnosis of PBC is readily reached by the detection of specific AMA directed against PDH-E2	PBC SS T1DM SLE RA	^60–62^
Creatine kinase S-type, mitochondrial (mtCK, UniProt:P17540)		
An enzyme that plays a role in the production of energy within the mitochondria	RA, T1DM	^63,64^
Dimethyladenosine transferase 2, mitochondrial (TRMT10C, UniProt:Q9H5Q4)		
Is involved in the transfer of methyl groups to specific adenosine residues in mitochondrial tRNAs. Catalyzes the dimethylation of adenosine at position 10 in mitochondrial tRNAs. This modification is important for the proper folding and function of the tRNAs, which are essential components in protein synthesis	MS RA	^64,65^
Cytochrome c1, heme protein, mitochondrial (UniProt:P08574)		
A component of the electron transport chain in mitochondria, which is responsible for generating ATP through oxidative phosphorylation. It is encoded by the nuclear DNA and synthesized in the cytoplasm before being imported into the mitochondria	AU RA T1DM	^63,64,66^

^1^Autoimmune diseases: *RA* rheumatoid arthritis, *AS* ankylosing spondylitis, *AIAS* autoimmune atherosclerosis, *PA* psoriatic arthritis, *AIT* autoimmune thyroiditis, *SS* Sjogren's syndrome, *PBC* primary biliary cholangitis, *T1DM* type 1 diabetes mellitus, *MS* multiple sclerosis, *AU* autoimmune uveitis, *SLE* systemic lupus erythematosus.

## Discussion

The present study aimed to explore several immune mechanisms that operate in the human body, where an external, frequently consumed environmental factor, namely mTG, might drive chronic diseases. The potential role of the microbial enzyme in CD induction^8,9,40–44,46–48,50,51^ and other autoimmune and neurodegenerative diseases was recently extensively described^6,7,22,26–28,39,46^. Various deleterious effects were attributed to this enzymatic food additive and corresponding pathogenic mechanisms were suggested^6–9,22,26–28,44–46^.

Six pairs of similar immunogenic sequences were detected between human endogenous antigens, derived from cross-reactive antibodies, and between mTG immune epitopes (Table 1). All of them showed a strong binding affinity to at least one of the HLA-I and HLA-II alleles and play a crucial role in cellular functions and body homeostasis (Table 2).

Reviewing those six similar pairs of proteins, a functional relationship to the mTG can be suggested:

1. *The fibrinogen alpha chain* is part of the coagulation system that joins factor XIII to establish an efficient clot. Factor XIII and mTG are integral members of the TG family^67^, both having the capacity to deamidate or transamidate acyl donors and acceptors molecules. There is no knowledge yet of on circulating mTG, nor its ability to coagulate, however, its intra-enterocytic transport and sub-epithelial deposition was documented^46^ and its relative resilience to oxidative compounds was recently reported^51^. Furthermore, restructured meat contains mTG and fibrinogen^68^, and fibrin gels crosslinked by a mTG are used in the industry, where the mTG reactions are comparable to those of factor XIII and tTG^69^. The potential pro-coagulant capacity of mTG is still an enigma.
2. *Histone H1.2* plays a pivotal role in chromatin and nucleosomes stability and functionality. Interestingly, cross-linking of histone by transglutaminase is well documented. Being a universal protein condenser, transglutaminase can modify core histone and regulate chromatin condensation, thus, impacting gene expression^70–73^. The cross-linking might result in free histone deprivation. In fact, epigenetic is a major pathway in ADs initiation and development, including in CD evolvement^72,73^.

The direct mTG action on histone 1.2 deserves more studies. The question arises whether during the intra-enterocytic transport, can mTG impact the gene expression of the human enterocyte?

3. *Dihydrolipoyllysine-residue acetyltransferase component of pyruvate dehydrogenase complex, mitochondrial* is an essential enzyme in mitochondrial energy metabolism and preservation. It appears that TG2 is important in mitochondrial functions and dysfunctions^74^. Upon activation, the enzyme can change the assembly of respiratory chain complexes and modulate the transcription of critical mitochondrial genes. In general, the bacterial enzyme imitates the functions of the human one; however, the impact of mTG on the mitochondrial energetic homeostasis remains to be disclosed.
4. *Creatine kinase S-type, mitochondria*. Creatine kinases represent a large family of isoenzymes that participate in intracellular energy homeostasis. Mitochondrial creatine kinase is responsible for the transfer of high energy phosphate to the cytosolic carrier, creatine. Creatine kinase S-type is a family member that plays a role in the mitochondrial energy metabolism and production, in organs with large, fluctuating energy demands, such as heart, skeletal muscle and brain^75^. Indeed, mitochondrial creatine kinase dysfunction was reported in heart, muscle and neurodegenerative conditions^76^. The observation that creatine reduces transglutaminase-catalyzed protein aggregation^77^ may connect various neurodegenerative diseases, like Alzheimer's, Parkinson's, and Huntington's diseases to creatine kinase dysfunction, reducing tissue creatine level, resulting in higher cross-linking activity of the local tTG^78^. Notably, mTG can functionally imitate the posttranslational modification executed by its family member, the tTG. However, the impact of mTG on those tissues is not yet known. Interestingly, abnormal energy metabolism was described in RA and T1DM^79,80^ (Table 2). In parallel, transglutaminase is implicated in both of the diseases. TG2 participate in synovial inflammation, bone erosion in RA, and in island cell dysfunction in T1DM^81–84^**.** Theoretically, if mTG reaches those target organs, comparable damage might be induced.
5. *Dimethyladenosine transferase 2, mitochondrial*. By the transfer of methyl groups to specific adenosine residues in mitochondrial tRNAs, this enzyme is essential for the proper folding and function of the tRNAs, which are essential components in protein synthesis. In fact, mitochondrial dysfunction exists in MS^85,86^ (Table 2) and in RA^79,80^ (Table 2) and both diseases are affected by tTG, as mentioned above, for RA^81–83^, but also in MS^87,88^. The place of the tTG functional imitator, namely the mTG, remain to be explored.
6. *Cytochrome c1, heme protein, mitochondrial* is integral and pivotal for the mitochondrial electron transport chain, responsible for generating ATP through oxidative phosphorylation. It represents a potential clinical marker for mitochondrial and cellular damage^89^. Mitochondrial failure, accompanied by inadequate energy supply and increased oxidative stress, exists in RA^79,80,90,91^ (Table 2), in T1DM^80,92^ (Table 2) and in AU^93^ (Table 2). Moreover, the mitochondrial Cytochrome c is affected in RA^94,95^, T1DN^96,97^ (Table 2) and in AU^98^ (Table 2). In parallel, the posttranslational modified ability of the tTG to cross-link mitochondrial protein^70–74,77,78^ and the mTG cross-linking capacity of cytochrome c, using its lysine residue as an acyl acceptor^99,100^, constitutes a confirmation of the tTG and mTG capacity to regulate mitochondrial proteins, thus contributing to this organelle dysfunction.

It can be summarized that tTG is involved in the regulation of the mitochondrial energy productive and regulatory machinery. This ubiquitous enzyme can cross-link histone, control chromatin condensation, determine gene expression^70^^,^^71^, affect mitochondrial functions^74^, its activity is affected by creatine kinases and by free creatine^78,79^ and its cross-linking activity can impact key essential mitochondrial molecules responsible for energy equilibrium in several autoimmune^81–84,87,88^ and neurodegenerative diseases^79^. In addition, the tTG is important in degradation of damaged mitochondria, thus playing as a gatekeeper of the mitochondrial functional homeostasis^101^.

In fact, a lot is still unknown as to whether mTG can replace tTG in all these activities. The fact that bacterial enzyme can cross the enteric epithelial lining^46,51^, have the capacity to cross-link proteins that contain acyl donors (glutamine) and acceptors (lysine)^8,9,22,26,27,37,45,47^, mount specific antibodies to its cross-linked complexes^7,9,40–43,45,47–49^ and be involved in initiation and progression of ADs, is an indication of its disadvantages, being a potential public health concern, and a caveat to public well-being^9,22,26,27,45^.

**Figure 3:**
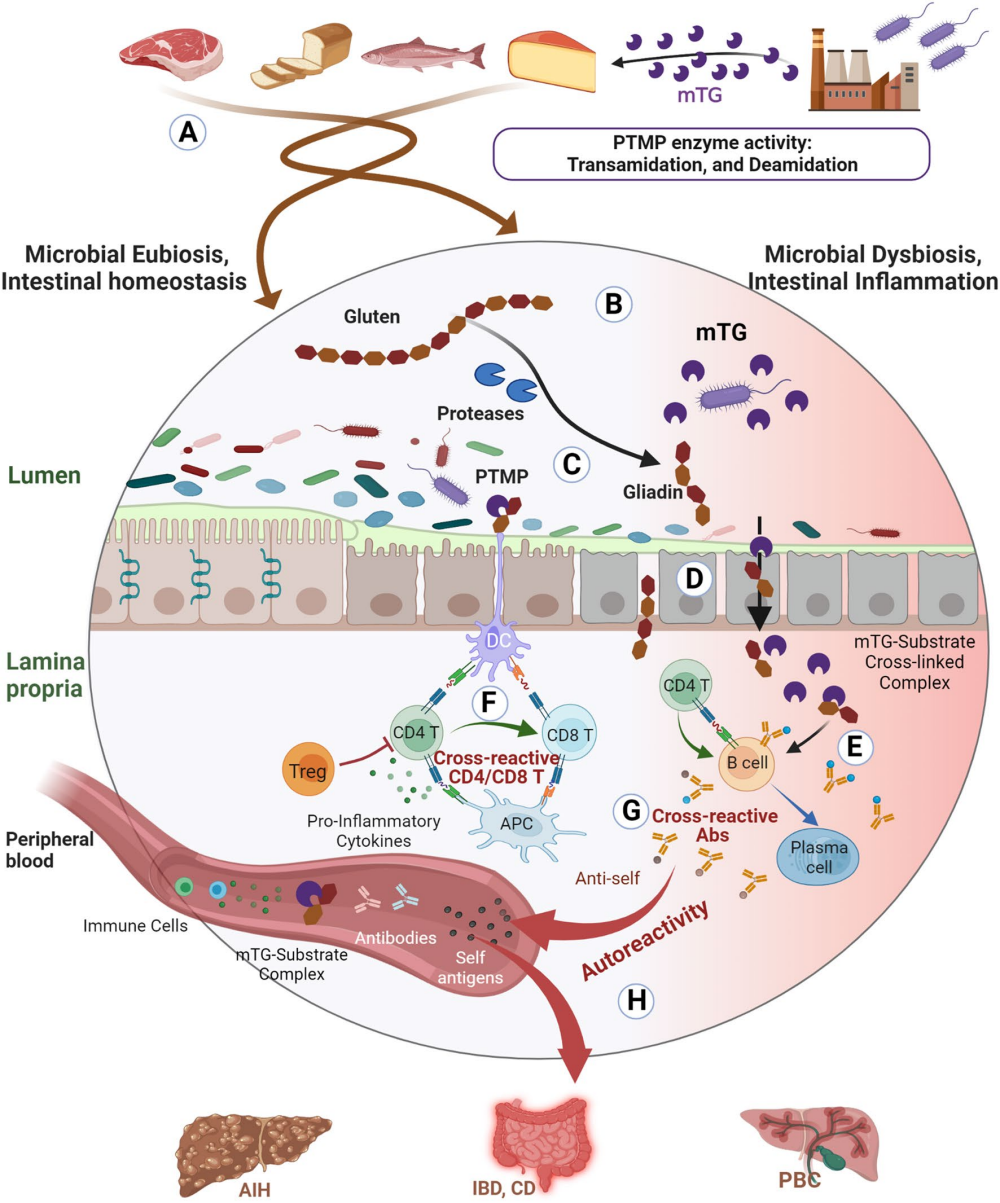
Schematic presentation of cross-reactivity and sequence similarity between mTG-Substrate complexes and gut-antigens that are associated with ADs. (**A**) Oral consumption of food products that were processed with mTG, such as meat, fish, dairy and bread. (**B**) mTG-substrate’ complexes, such as mTG-gliadins, reach the gut lumen. (**C**) Gliadins, and other processed food products, are a substrate for mTG cross-linkage, turning a naïve molecule to immunogenic one. The result is an increase in mtg-induced PTMP that human digestive enzymes cannot break down, thus, inducing gut inflammation and damage to the intestinal epithelium. (**D**) mTG can potentially damage the lining mucus by breaking its stability and compromising tight junction functional integrity. mTG-Gliadin and other mTG complexes might penetrate into the lamina propria through open junctions or trans-enterocytically. (**E**) In the lamina propria, mTG-cross-linked complexes induce pro-inflammatory cytokines that drive T cells and B cells activation. (**F**) CD4 T cells initiate an immune response against mTG-PTMP after APC presentation of epitopes on HLA-II. CD8 T cells can be activated when they are exposed to epitopes presented on HLA-I, and activated by CD4 T cells. Cross-reactivity at the T cell level involves recognition of certain mTG-PTMP epitopes which are similar to self-epitopes. (**G**) Cross-reactivity at the B cell level when clonal antibodies bind to mTG-PTMP epitopes that are similar to self-epitopes. (**H**) Autoreactive antibodies, effector B and T cells, and mTG-substrate complexes travel through blood vessels to peripheral organs. They can potentially become autoreactive when they encounter similar self-epitopes, and an autoimmune response will be directed against the host as well.

The current study brings, for the first time, two new potential pathogenic pathways: (1) relating the mTG enzyme to autoimmune and other chronic human conditions; (2) cross-reactive antibodies and sequence similarity between the environmental enzyme and endogenous human self-antigens. To these two pathogenic mechanisms the epitope sharing between the environmental gluten/gliadin peptides and multiple human antigens should be added. Intriguingly, gluten/gliadin structural segments are prime substrates for mTG de/transamidation^6–9,22,39–46^. This posttranslational modification is operating in the processed food industries, in bakeries and more importantly, in the human gut lumen^8,9,22,26,27,45^. It seems that the mTG-gluten-human self-epitopes axis is interactive and auto-immunogenic. Those three interrelated pathways are the basis for our current novel hypothesis, whereby, two very common environmental domains, plants and microbes, and gluten and mTG, respectively, are joining together to induce autoimmunity and other gluten-dependent inflammatory diseases^4,7,9,22,26,27,32,39,41^. Interestingly, gluten avoidance was recently reported to alleviate symptoms and disease activity of non-celiac ADs^81,82,102–106^, although, gluten withdrawal is not devoid of side effects^107–109^. Taken together, both external factors, the mTG and gluten-containing nutrients, can operate as the mythological Trojan horse to drive luminal and extra-intestinal ADs^29^. Figure 3 presents schematically the cross-reactivity and sequence similarity between mTG-Substrate complexes and gut-antigens that are associated with ADs.

The list of side effects of the processed food additive, mTG, and its cross-linked complexes is constantly expanding^6–9,22,39,41–45^. Multiple mechanisms were offered for those health-targeted detrimental effects. The mTG compromises tight junctional functional integrity, enhancing a leaky gut syndrome^9,22,45^ and enhances enteric epithelial gliadins uptake and transportation^8,9,22,45,105,110^. The foreign molecules, mTG and gliadin, are trans-enterocyticaly transported to face and challenge the sub-epithelial immune systems^46^. The microbial enzyme can compromise the mechanical intestinal protective barriers by introducing resistant isopeptide bonds, thus, perturbating mucin fluidity and stability, resulting in enhanced attachment of pathogenic luminal germs or other harmful factors to the epithelial receptors^111^. More so, it suppresses mucosal and systemic immune systems. Indeed, *Streptococcus suis*-originated mTG exerts anti-phagocytic effect, resulting in suppressing a major immune protective barrier^112–115^. As a bacterial survival factor, suppressing gut immunity, the mTG is a growth factor for luminal microbiota, dysbiota and pathobionts, as was reported in *Lactococcus* strain^116,117^. The problem is accentuated since more sophisticated bioengineered technics produce higher yield and more active mTG for industrial usage^118–120^. The enzyme represents a double-edged sword, a protective bacterial factor in the gut lumen, hence, a human hostile one, compromising human health^45^. In view of the active horizontal gene transfer in the gut lumen^28^, a major question arises. Can the harmful mTG be laterally transferred to the physiological microbiome, as is happening for the bacterial resistant genes spread?^121,122^. On the same line, recently, the trans-membranal region of mTG was suggested to participate in the recognition of host's immune signals and reciprocal bacterial communication, by binding to its corresponding ligand^123^.

The cross-reactive antibodies warrant some clarification. Polyclonal antibodies contain a heterogenous mixture of antibodies produced by different clones of plasma B cells against different epitopes of a whole antigen, whereas monoclonal antibodies are a homogenous population of antibodies that are produced by a single clone of B cells. Thus, polyclonal antibodies interact with different epitopes on a single antigen, while monoclonal antibodies interact with a particular epitope on the same antigen. This may explain the reactivity of anti-mTG polyclonal antibody with 18 out of 77 autoantigens and the reactivity of anti-mTG monoclonal antibody with only 9 out of 77 human tissue antigens^124^.

These are some strengths of the current study. It combines the human to the mTG epitopes, applying two methods, namely, cross-reactive antibodies and sequence similarity. It describes two members of the transglutaminase's family that shares comparable functions. The environmental mTG has a much broader substrate activity than its endogenous tTG one. So, theoretically, it might cross-react with more human antigens.

As for the study’s limitations, the major one is the lack of proof that the mTG itself or its post-translated modified proteins and cross-linked complexes can circulate systemically to reach peripheral target organs. However, the fact that when active mTG is abandoned in the gut lumen, it reaches the baso-lateral compartment of the enterocytes and its cross-linked complexes are immunogenic, strengthen the present hypothesis. In addition, the presented findings are limited to the curated epitopes that are currently found in the Immune Epitope Database (IEDB, https://www.iedb.org), and to 77 different human tissue antigens that were tested for cross-reactivity. Yet it provides an indication of such antigens that can potentially provoke molecular mimicry.

To further substantiate the present working hypothesis and strengthen the cause-and-effect relationship between the cross-reactive antibodies against mTG in patients with various ADs, those purified antibodies should be checked in the autoimmune affected patient's sera and passively transferred to appropriate animal models. We hope that the present observations will encourage further studies to establish causality between cross-reactivity and sequence similarity and the corresponding ADs.

## Methods

### Cross-reactive antibodies

To demonstrate cross-reactivity between mTG and various human target tissue antigens, the steps for the ELISA method were extracted from various manuscripts published by Vojdani et al.^4,32,125–131^.

In brief, mouse monoclonal antibody to recombinant mTG (MyBioSource, England) and affinity-purified rabbit polyclonal antibodies made against mTG (Zedira GmbH, Germany) were applied to 77 different human tissue antigens using the ELISA method. Different wells of ELISA plates were coated with various tissue antigens representing these categories: coagulation and heart, joints, diabetes-related, skin, epithelial and tight junctions, liver, lung, thyroid, the nervous system, and cellular antigens. The complete list of their source antigens and optimal concentrations is shown in Table S1 in the Supplement section.

Each antigen was first dissolved in 0.01 M of PBS at pH 7.4, then further diluted in carbonate buffer pH 9.5, in optimal amounts ranging from 0.5 to 2 µg per 100 µl, and was added to duplicate wells. Following incubation for 12 H at 25 °C, and an additional 12 H at 4 °C, the plates were washed 5 times, after which 200 µl of blocker containing 1% bovine serum albumin and 1% dried milk was added to each well. After a repeat incubation and washing, mouse monoclonal antibody against mTG, at a dilution of 1:200 and polyclonal antibody at a dilution of 1:400 was added to different sets of ELISA wells, coated with human tissue antigens. Plates were incubated for 1 H at 25 °C, and after another washing, 100 µl of alkaline phosphatase goat anti-mouse IgG at a dilution of 1:600 and goat anti-rabbit, at a dilution of 1:800, were added to different sets of ELISA plates. After another repeat of incubation and washing, 100 µl of substrate was added to each well, and color development was measured at 405 nM.

### Sequence similarity

In search of immunoreactive epitopes, all human epitopes that relate to ADs were obtained from IEDB^132,133^. The IEDB was searched with the following keywords: Epitope: “Linear peptide”, Epitope Source: “Human Organism”, Host: “Human”, Assays included: “T cells”, “B cells”, “HLA-I”, “HLA-II”, Outcome: “Positive Assays”, and Disease: “Autoimmune”. About 67,000 epitopes were extracted from in-vivo experimental studies as antigens implicated with at least one of 61 ADs categories. The complete sequence of mTG protein, Uniprot: P81453, Organism: Streptomyces Mobaraensis, was acquired from the UniProt Knowledgebase (https://www.uniprot.org/)^134^.

As for sequence alignment, a Pairwise Local Alignment tool, EMBOSS Matcher^135,136^ was employed to explore sequence similarity between the aggregated human auto-epitopes and the mTG protein. This tool searches for local similarities between any two sequences by implementing an algorithm based on Bill Pearson's Lalign application, version 2.0u4 (Feb. 1996). A cutoff was applied on EMBOSS Matcher’s results to identify those epitopes that could have a higher probability of inducing molecular mimicry. The aligned peptides’ cut-off was kept at a minimum of seven identical AAs, and at peptide length > 12 AAs.

All human epitopes were captured in IEDB from experimental assays published in scientific literature. However, mTG sequences that were extracted and identified by EMBOSS Matcher required additional analysis to assess their immunological potential reactivity. IEDB Immunogenicity Prediction services offer tools to analyze the binding affinity values in terms of half maximal inhibitory concentration (IC50) of peptides binding to HLA-I/II alleles and to assess their potential to elicit an immune response. These tools were utilized to filter out all mTG sequences that were not considered to have immunogenic potential.

**Figure 4:**
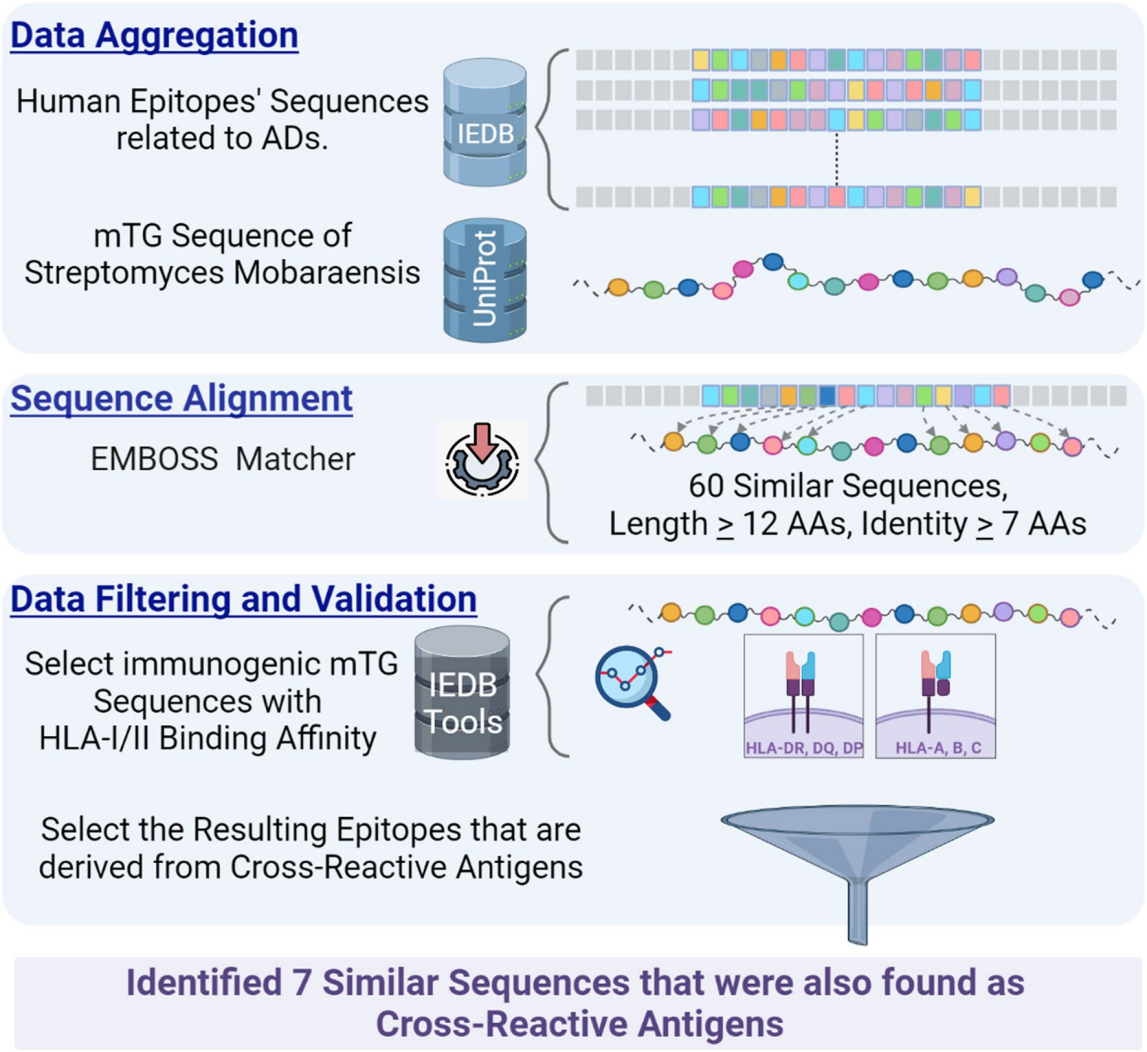
A graphical representation of the workflow for searching sequence similarity. *Data Aggregation*: human epitopes that are implicated in ADs were extracted from IEDB and UniProt was searched to retrieve mTG protein sequence. *Sequence Alignment*: Emboss Matcher was employed; 60 similar sequences were found with a cut-off of at least 7 identical AAs and peptide length > 12 AAs. *Data Filtration and Validation*: IEDB analysis tools were employed to validate those mTG sequences that are immunogenic, and have HLA-I/II binding affinity. Out of those results, 7 similar sequences were of human antigens that were previously identified to cross-react with mTG protein.

As a final selection, the resulting similar sequences were cross-checked with the cross-reactive antigens, and the concluding list includes human epitope sequences that are derived from those antigens. The methodology is presented as a flowchart in Fig. 4.

## Conclusion

In summary, our findings support the potential contribution of mTG to various autoimmune diseases, which should be the subject of future studies. The presented shared cross-reactive antibodies and sequence similarity between the mTG and human immune epitopes, presents two novel pathological mechanisms that might compromise public health. It is hoped that the current findings will encourage future exploration of the mTG-human enigma.

### Supplementary Information

Supplementary Table S1.

## Data Availability

The data and software that supports the findings of this study are openly available in: The Immune Epitope Database (IEDB) at www.iedb.org, reference^132,133^. UniProt Knowledgebase www.uniprot.org, reference^134^. Pairwise Local Alignment tool, EMBOSS Matcher, at www.emboss.sourceforge.net, reference^135,136^, A python script can be found at https://raw.githubusercontent.com/ebi-wp/webservice-clients/master/python/emboss_matcher.py.

## Abbreviations

ADs: Autoimmune diseases
ASIA: Autoimmune/inflammatory syndrome induced by adjuvants
GIT: Gastrointestinal tract
mTG: Microbial transglutaminase
tTG: Tissue transglutaminase
CD: Celiac disease
AAs: Amino acids
OD: Optical density
