# Supplementary material for: Cross-reactivity and sequence similarity between microbial transglutaminase and human tissue antigens
Source: Sci Rep. 2023 Oct 16;13:17526. doi: 10.1038/s41598-023-44452-5 (PMC10579360; doi:10.1038/s41598-023-44452-5)
Supplement: Supplementary file 1 — Supplementary Table S1. [file 41598_2023_44452_MOESM1_ESM.pdf]

SUPPLEMENTAL TABLE S1: ANTIGENS, ABBREVIATIONS, CONCENTRATIONS AND SOURCES

**Coagulation & Heart-Associated Autoantigens**	**Conc***	**Source**
Heparin	2	Sigma
Cardiolipin	1	Sigma
Fibrinogen	2	Sigma
Alpha-myosin	1	EMD Bioscience
Tropomyosin	0.5	Sigma
Platelet glycoprotein (PG)	2	Bio-Synthesis
Beta-2 glycoprotein A	1	Bio-Synthesis
**Joint- Associated Autoantigens**	**Conc**	**Source**
Collagen	1	Sigma
Fibulin	1.5	Bio-Synthesis
Human Ro60	1	Bio-Synthesis
Alpha-enolase	2	Bio-Synthesis
Citrullinated alpha-enolase	1	Bio-Synthesis
Chondroitin sulphate (CS)	1	Sigma
**Diabetes- Associated Autoantigens**	**Conc**	**Source**
Insulin	1	Sigma
Insulin receptor (Insulin R)	2	Bio-Synthesis
Islet cell autoantigen (ICA)	1	Bio-Synthesis
Glutamic acid decarboxylase 65 (GAD-65) & GAD-67	2	Bio-Synthesis
Zinc transporter isoform 8 (ZnT8)	1	Bio-Synthesis
**Skin- Associated Autoantigens**	**Conc**	**Source**
Human epidermal keratin (HEK)	1	Sigma
Tyrosinase	1	Sigma
Desmoglein-3 E-cadherin (DEC)	2	Novus Biologicals
Transglutaminase 3 (TG3)	2	Bio-Synthesis
**Epithelial & Epidermal Tight Junction Protein**	**Conc**	**Source**
Zonulin & Occludin	2	Abcam
DPP IV	1	Sigma
Beta-catenin	1	Bio-Synthesis
Epithelial cell antigen (ECA)	1	Sigma
Translgutaminase 2 (TG2)	2	Sigma
Calprotectin	0.5	Bio-Synthesis
Neutrophil cytoplasmic antigen	1	Bio-Synthesis
**Biomarkers of Autoimmunity**	**Conc**	**Source**
Anti-nuclear antigen (ANA)	O	INOVA
Extractable nuclear antigen (ENA)	O	INOVA
Double-stranded DNA (dsDNA)	O	INOVA
Actin	O	INOVA
Mitochondrial M2	O	INOVA
Rheumatoid factor (RF)	O	INOVA
**Lung- Associated Autoantigens**	**Conc**	**Source**
Lung surfactant protein-C	2	Bio-Synthesis
Epithelial sodium channel alpha	2	Bio-Synthesis
Claudin-7.1 & 7.2	1.5	Bio-Synthesis

**Nervous System- Associated Autoantigens**	**Conc**	**Source**
Myelin basic protein	2	Sigma
Myelin oligodendrocyte glycoprotein	2	Bio-Synthesis
Neurofilament protein	2	Bio-Synthesis
Ganglioside GM_1	0.5	Sigma
Ganglioside GM_2	0.5	Sigma
Sulphatide	1	Sigma
Transglutaminase 6 (TG6)	2	Bio-Synthesis
Enteric nerve	2	Bio-Synthesis
Alpha-synuclein & Beta-synuclein	1	Bio-Synthesis
Synapsin	1	Bio-Synthesis
Brain-derived neurotrophic factor (BDNF)	2	Bio-Synthesis
Somatotropin	2	Sigma
Phosphorylated tau protein	2	Bio-Synthesis
Amyloid-β peptide (AbP)	2	Bio-Synthesis
Claudin-5	1	Bio-Synthesis
Acetylcholine receptor	2	Bio-Synthesis
Dopamine D_1 receptor (D1 R)	2	Bio-Synthesis
Dopamine D_2 receptor (D2 R)	2	Bio-Synthesis
N-methyl-D-aspartate receptor (NMDAR)	2	Bio-Synthesis
Rabaptin-5 (Rab-5)	1	Bio-Synthesis
Cerebellar	1	Bio-Synthesis
Aquaporin-4 (AQP4)	2	Bio-Synthesis
S100B	1	EMD Bioscience
Glial Fibrillary Acidic Protein (GFAP)	2	Bio-Synthesis
Glutamate receptor 1,5	1	Bio-Synthesis
Amphiphysin	1	Bio-Synthesis
Gamma-aminobutyric acid receptor (GABA-R)	1	Bio-Synthesis
Glycine receptor	1	Bio-Synthesis
Thyrosine kinase	1	Bio-Synthesis
Fatty acid binding protein 7	0.5	Bio-Synthesis
Proteolipid protein	0.5	Bio-Synthesis
Alpha B-crystallin	1	Bio-Synthesis
**Thyroid-Associated Autoantigens**	**Conc**	**Source**
Thyroglobulin	1	EMD Bioscience
Thyroid peroxidase (TPO)	0.5	Bio-Synthesis
Thyroid stimulating hormone (TSH)	1	Sigma
Thyroid stimulating hormone receptor (TSHR)	1	Sigma
Triiodothyronine (T3)	1	Sigma
Thyroxine (T4)	1	Sigma
Myeloperoxidase	1	Sigma
**Liver- Associated Autoantigens**	**Conc**	**Source**
Liver microsomal antigen (LMA)	0.5	Sigma

*Concentration (Conc) is in microgram per well of ELISA plate
